# Superiority of the triglyceride glucose index over the homeostasis model in predicting metabolic syndrome based on NHANES data analysis

**DOI:** 10.1038/s41598-024-66692-9

**Published:** 2024-07-05

**Authors:** Haiyan Wan, Hongyi Cao, Peng Ning

**Affiliations:** Department of Endocrine and Metabolism, Chengdu Fifth People’s Hospital (The Second Clinical Medical College, Affiliated Fifth People’s Hospital of Chengdu University of Traditional Chinese Medicine), Geriatric Diseases Institute of Chengdu/Cancer Prevention and Treatment Institute of Chengdu, Chengdu, 611137 China

**Keywords:** Triglyceride-glucose index, Homeostasis model assessment, Metabolic syndrome, NHANES, Metabolic syndrome, Public health

## Abstract

The triglyceride-glucose (TyG) index is a simple and inexpensive new marker of insulin resistance that is being increasingly used for the clinical prediction of metabolic syndrome (MetS). Nevertheless, there are only a few comparative studies on its predictive capacity for MetS versus those using the traditional homeostasis model assessment (HOMA). We conducted a cross-sectional study using a database from the National Health and Nutrition Examination Survey (1999 March to 2020 pre-pandemic period). Using statistical methods, we compared the predictive abilities of the TyG index and HOMA (including HOMA of insulin resistance [HOMA-IR] and HOMA of beta-cell function [HOMA-β]) for MetS. A total of 34,195 participants were enrolled and divided into the MetS group (23.1%) or no MetS group (76.9%) according to the International Diabetes Federation (IDF) diagnostic criteria. After applying weighted data, the baseline characteristics of the population were described. Following the exclusion of medication influences, the final count was 31,304 participants. Receiver operating characteristic curve analysis revealed that while distinguishing between MetS and no MetS, the TyG index had an area under the curve (AUC) of 0.827 (sensitivity = 71.9%, specificity = 80.5%), and the cutoff was 8.75, slightly outperforming HOMA-IR (AUC = 0.784) and HOMA-β (AUC = 0.614) with a significance of *P* < 0.01. The prevalence of MetS in the total population calculated using the TyG index cutoff value was 30.9%, which was higher than that reported in the IDF diagnostic criteria. Weighted data analysis using univariate and multivariate logistic regression displayed an independent association between elevated TyG and HOMA-IR with the risk of MetS. Subgroup analysis further revealed differences in the predictive ability of the TyG index among adult populations across various genders and ethnicities, whereas such differences were not observed for children and adolescents. The TyG index is slightly better than HOMA in predicting MetS and may identify more patients with MetS; thus, its applications in a clinical setting can be appropriately increased.

## Introduction

As a complex and diverse health issue, metabolic syndrome (MetS) has become the focus of public health worldwide. Symptoms including obesity, hypertension, hyperglycemia, and dyslipidemia greatly increase the risk of cardiovascular disease, diabetes, and other chronic diseases and have a profound impact on the health of individuals as well on society^1^. Thus, there is an urgent requirement to identify effective methods to accurately predict MetS. In this field, the ability of the triglyceride-glucose (TyG) index, a new marker of insulin resistance, has attracted much attention in recent years to evaluate MetS^2–4^. The TyG index is a simple and inexpensive metabolic index that can be calculated by combining triglyceride and glucose levels and is considered to reflect pancreatic function and insulin resistance in tissues^5^. In contrast, homeostasis model assessment (HOMA) is a traditional indicator used to determine insulin resistance and insulin sensitivity. HOMA mainly includes HOMA of insulin resistance (HOMA-IR), HOMA of insulin sensitivity (HOMA-IS), and HOMA of beta-cell function (HOMA-β), and is obtained by calculating the relationship between fasting insulin levels and blood glucose levels. However, HOMA has some limitations in predicting MetS such as not accurately reflecting the effect of triglycerides. Moreover, comparative studies on the predictive values of these two indicators in MetS are still relatively limited. At the time of writing this article, only Son et al. had reported that the TyG index was significantly better than HOMA-IR in a study of Korean adults^6^, and Dundar et al. had found that the TyG index in girls was better than HOMA-IR in a study involving Turkish children^7^. However, the sample sizes of these studies were small.

Therefore, we compared and analyzed the performance of the TyG index and HOMA in predicting MetS using large-scale population data from the National Health and Nutrition Examination Survey (NHANES) from 1999 to March 2020 (pre-pandemic period). There is an urgent need for a more comprehensive understanding of the advantages and limitations of these two indicators and their practical application values in clinical practice. Through this study, we hope to provide more accurate and reliable tools for the screening and management of MetS at an early stage, thus helping to reduce the incidence of MetS-related chronic diseases and improving overall health. Therefore, the results of this study may have a certain impact on future medical practice and public health policy-making.

## Materials and methods

### Study population

**Figure 1:**
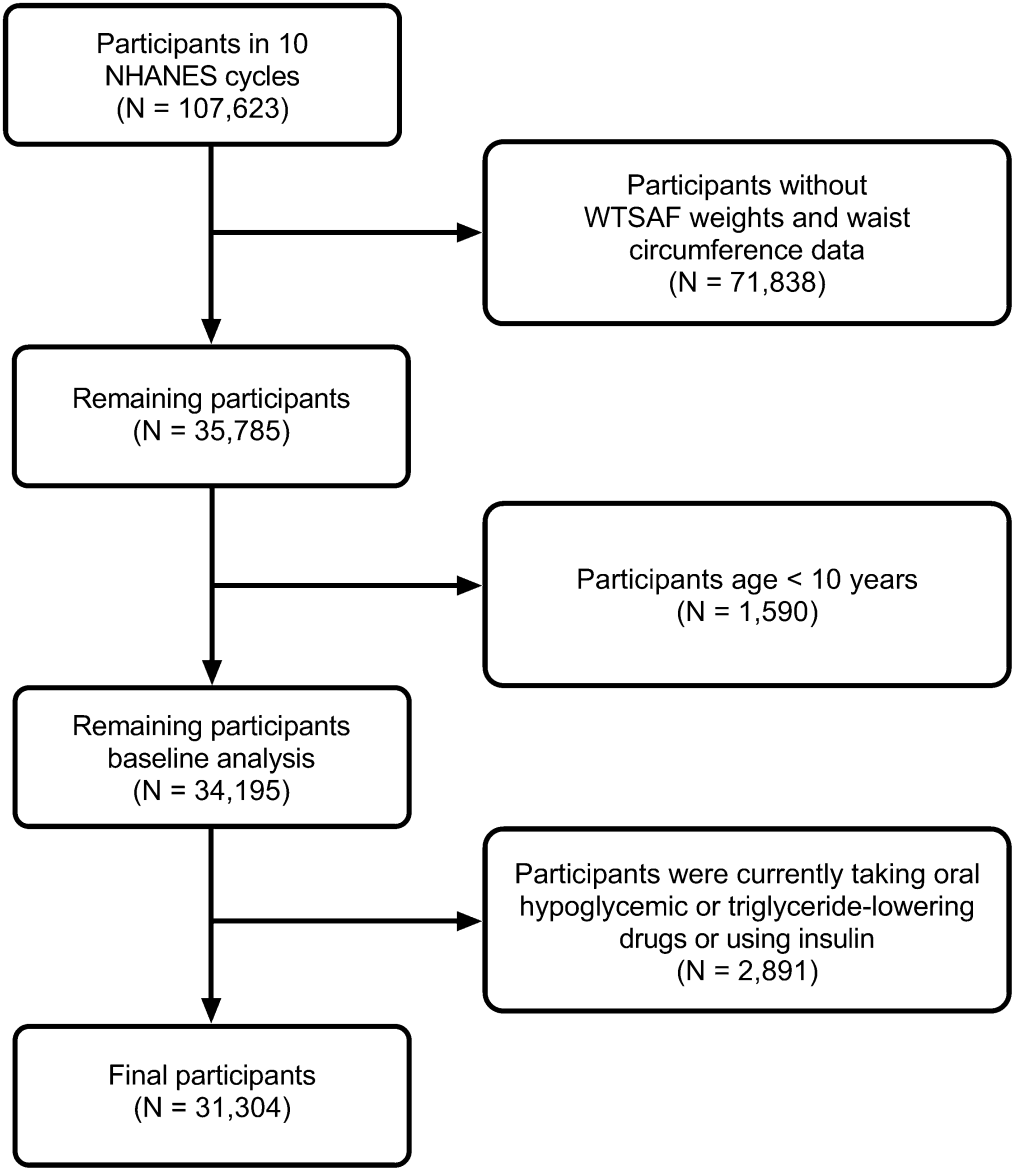
Participant flowchart. Abbreviations: NHANES, National Health and Nutrition Examination Survey.

This study utilized data from NHANES in the United States. All databases are accessible through the NHANES website (https://wwwn.cdc.gov/nchs/nhanes/Default.aspx). NHANES is a national survey conducted by the Centers for Disease Control and Prevention to assess the health and nutritional status of adults, children, and adolescents in the United States through a complex, stratified, random-sampling method, allowing estimations to be generalized to the entire United States population. We analyzed data from the past 10 cycles (1999 to March 2020 pre-pandemic period), which included a total of 107,623 participants. Among them, 71,838 participants lacking waist circumference data or WTSAF weights were excluded from the study. Additionally, 1590 participants < 10 years of age were excluded due to the absence of a clear definition for MetS diagnosis, and the remaining 34,195 participants were analyzed at baseline. Finally, 2891 participants currently taking oral hypoglycemic or triglyceride-lowering medications or those using insulin were excluded. Thus, a final cohort of 31,304 individuals was used to assess the diagnostic value of the TyG index and HOMA for MetS. The participant flowchart is depicted in Fig. 1.

### Calculation formulae

The TyG index was mainly calculated using blood glucose and triglyceride levels as follows:

TyG index = Ln [fasting triglyceride (mg/dL) × fasting blood glucose (mg/dL) / 2]^8^; HOMA mainly included HOMA-IR, HOMA-IS, and HOMA-β, and the formulae for calculation were as follows:

HOMA-IR = fasting blood glucose (mmol/L) × fasting insulin (μU/mL)/22.5;

HOMA-IS = 1/HOMA-IR = 22.5 / fasting blood glucose (mmol/L) × fasting insulin (μU/mL);

HOMA-β = 20 × fasting insulin (μU/mL) / (fasting blood glucose [mmol/L] – 3.5)^9^.

### Definition criteria

The following diagnostic criteria of the International Diabetes Federation (IDF) 2007 for MetS were adopted: 1. Central obesity; 2. Abnormality in any two or more of the four indicators: blood pressure, blood glucose, triglyceride, and high-density lipoprotein cholesterol. However, the criteria for adults^10^ and children and adolescents^11^ were different. The diagnostic criteria for adult MetS are shown in Table 1 and those for children and adolescents in Table 2. The percentage waist circumference for children and adolescents is shown in Supplementary Table S1. Questionnaire data for smoking, drinking, and education: As NHANES did not have questionnaires for smoking and alcohol consumption for children and adolescents, smoking and alcohol consumption data were collected only for individuals aged 20 years and older. “Smoked at least 100 cigarettes in life” was defined as smoking, and “Had at least 12 alcohol drinks per year” was defined as drinking. “Less than 9th grade” and “9th–11th grade” were defined as education below high school level; “High School Grad/GED or Equivalent” was defined as High School education level; and “Some College or AA degree” and “College Graduate or above” were defined as education above high school level. The definition of diabetes for all participants was based on the American Diabetes Association 2023 standard^12^ combined with questionnaires, and if they met any of the following criteria: “Have been informed of diabetes,” “Fasting blood glucose (FBG) levels ≥ 126 mg/dL” or “Glycosylated hemoglobin (HbA1c) levels ≥ 6.5%.” The definition of hypertension for all participants was based on the American Heart Association/American College of Cardiology 2017 standard^13^ combined with questionnaires, and if they met any of the following criteria: “Had been told about hypertension,” “Systolic blood pressure (SBP) ≥ 130 mm Hg,” or “Diastolic blood pressure (DBP) ≥ 80 mm Hg.”

**Table 1:** IDF consensus definition of metabolic syndrome in adults.

According to the adult IDF definition, for a person to be defined as having the metabolic syndrome they must have: Central obesity (defined as an raised waist circumference, in the USA it is more than 102 cm for men and more than 88 cm for women.) plus any two of the following four factors:	
Raised triglycerides	≥ 150 mg/dL (1.7 mmol/L) or treatment for this lipid abnormality
Reduced high-density lipoprotein cholesterol	< 40 mg/dL (1.03 mmol/L) in males < 50 mg/dL (1.29 mmol/L) in females or treatment for this lipid abnormality
Raised blood pressure	SBP ≥ 130 or DBP ≥ 85 mm Hg or treatment of previously diagnosed hypertension
Raised fasting blood glucose	FPG ≥ 100 mg/dL (5.6 mmol/L), or treatment of previously diagnosed type 2 diabetes

*IDF* international diabetes federation, *SBP* systolic blood pressure, *DBP* diastolic blood pressure, *FBG* fasting blood glucose.

**Table 2:** IDF consensus definition of metabolic syndrome in children and adolescents.

Age group (years)	Central obesity (WC)	Triglycerides	HDL-C	Blood pressure	Glucose (mmol/L) or known T2DM
6– < 10	≥ 90th percentile	Metabolic syndrome cannot be diagnosed, but further measurements should be made if there is a family history of metabolic syndrome, T2DM, dyslipidemia, cardiovascular disease, hypertension and/or obesity			
10– < 16 Metabolic syndrome	≥ 90th percentile or adult cut-off if lower	≥ 1.7 mmol/L (≥ 150 mg/dL)	< 1.03 mmol/L (< 40 mg/dL)	Systolic ≥ 130/diastolic ≥ 85 mm Hg	≥ 5.6 mmol/L (100 mg/dL) (or known T2DM)
16 + Metabolic syndrome	Use existing IDF criteria for adults				

*IDF* international diabetes federation, *WC* waist circumference, *HDL-C* high-density lipoprotein cholesterol, *T2DM* type 2 diabetes mellitus.

### Statistical analysis

R software version 4.3.1 (R Core Team, Vienna, Austria) was used for data entry and processing. Packages such as “survey,” “weights,” “matrix,” and “pROC” were mainly selected. GraphPad Prism, version 9.4.1 (GraphPad Software, San Diego, CA, USA) was used to create images. First, fasting subsample weights were selected for the weighted processing of the selected data. During the consolidation of various cycles, “4/21.2 × WTSAF4YR” was chosen as the weight for the cycles from 1999–2002, whereas for the cycles from 2003 to 2016, with its 7 cycles, “2/21.2 × WTSAF2YR” was assigned as the weight for each cycle. For the period from 2017 to March 2020 (pre-pandemic cycle), “3.2/21.2 × WTSAFPRP” was selected as the weight. N represents the included population and WN represents the number of weighted representative American population. Continuous variable data are represented as mean ± standard error (SE), and categorical variables are represented as % (SE). *T*-test was used for continuous variables and Chi-square test was used for classification variables. The TyG index, HOMA-IR, and HOMA-β were transformed into dichotomous variables. After data weighting, univariate and multivariate Models 1, 2, 3, and 4 were established for logistic regression analysis. Then, the TyG index and the ability of HOMA-IR and HOMA-β to diagnose MetS were analyzed and evaluated using the receiver operating characteristic (ROC) curve, and the corresponding areas under curve (AUCs), sensitivity, and specificity were calculated. Lastly, subgroup univariate analysis was conducted for adults, children, and teenagers after data weighting based on population characteristics. As HOMA-IS is the reciprocal of HOMA-IR and its diagnostic efficiency is consistent with that of HOMA-IR, further logistic regression analysis and ROC analysis were not performed. α = 0.05 was considered statistically significant.

### Ethics approval and consent to participate

The NHANES data used in this study are public data and do not contain any personally identifiable information; thus, additional ethical review was not required. We have strictly abided by NHANES data usage specifications and privacy protection policies to ensure compliance and ethical data use.

## Results

### Baseline characteristics

The statistics of the data-completion ratio are shown in Table 3. We first divided 34,195 participants into 2 groups based on the IDF diagnostic criteria as follows: 26,304 (76.9%) in the no MetS group and 7,891 (23.1%) in the MetS group. The prevalence of MetS was 29.1% in adults and 4.8% in children and adolescents. After data were weighted for baseline analysis, the 2 groups of data were compared (Table 4). In the comparison of the TyG index and HOMA, to avoid the interference of drugs, 2,891 participants who were currently taking oral hypoglycemic and lipid-lowering drugs or using insulin were excluded. Finally, 31,304 participants were included in the analysis and were classified as 22,846 adults and 8,458 children or adolescents based on their age. Baseline analysis and intergroup comparison were conducted for each group. The baseline characteristics of adults are shown in Table 5 and those of children and adolescents in Table 6.

**Table 3:** Statistics of data-completion ratio.

Variable	N	Completion ratio (%)
Total	34,195	100
Gender	34,195	100
Age	34,195	100
Race	34,195	100
Education*	25,664	75.1
Smoking*	25,670	75.1
Drinking*	23,624	69.1
Diabetes	34,195	100
Hypertension	33,699	98.6
BMI	34,072	99.6
WC	34,195	100
SBP	31,090	90.9
DBP	31,090	90.9
HbA1c	32,109	93.9
FBG	31,952	93.4
INS	31,322	91.6
TG	31,822	93.1
TC	32,068	93.8
HDL-C	32,067	93.8
LDL-C	30,814	90.1
TyG	31,354	91.7
HOMA-IR	31,280	91.5
HOMA-IS	31,280	91.5
HOMA-β	31,220	91.3

*BMI* body mass index, *WC* waist circumference, *SBP* systolic blood pressure, *DBP* diastolic blood pressure, *HbA1c* glycosylated hemoglobin, *FBG* fasting blood glucose, *FINS* fasting insulin, *TG* triglyceride, *TC* cholesterol, *HDL-C* high density lipoprotein cholesterol, *LDL-C* low density lipoprotein cholesterol, *TyG* triglyceride-glucose, *HOMA-IR* homeostasis model assessment of insulin resistance, *HOMA-IS* homeostasis model assessment of insulin sensitivity, *HOMA-β* homeostasis model assessment of beta-cell function.

*Only adults who were 20 years and older were counted.

**Table 4:** Baseline characteristics of the general population.

Variable	All (N = 34,195)		MetS (N = 7891)		no MetS (N = 26,304)		*P*-value
	WN	mean ± SE or % (SE)	WN	mean ± SE or % (SE)	WN	mean ± SE or % (SE)	
Age, year	247,113,167	42.4 ± 0.2	62,475,040	51.8 ± 0.3	184,638,127	39.2 ± 0.2	< 0.01
Gender	247,113,167		62,475,040		184,638,127		
Male, %		48.7 (0.3)		45.2 (0.8)		49.9 (0.4)	< 0.01
Female, %		51.3(0.3)		54.9 (0.8)		50.1 (0.4)	< 0.01
Race	247,113,167		62,475,040		184,638,127		
Mexican American, %		8.9 (0.6)		8.8(0.7)		9.0 (0.5)	0.68
Other Hispanic, %		5.8 (0.5)		5.4(0.6)		5.9 (0.5)	0.16
Non-Hispanic White, %		66.5 (1.0)		70.8(1.2)		65.1 (1.0)	< 0.01
Non-Hispanic Black, %		11.6 (0.6)		9.6(0.6)		12.2 (0.6)	< 0.01
Other Race, %		7.2 (0.3)		5.4(0.4)		7.8 (0.4)	< 0.01
Diabetes	247,113,167		62,475,040		184,638,127		
Yes, %		10.9 (0.3)		27.2 (0.8)		5.4 (0.2)	< 0.01
No, %		89.1 (0.3)		72.8 (0.8)		94.6 (0.2)	< 0.01
Hypertension	245,383,671		62,409,339		182,974,332		
Yes, %		43.8 (0.5)		75.0 (0.8)		33.2 (0.5)	< 0.01
No, %		56.2 (0.5)		25.0 (0.8)		66.8 (0.5)	< 0.01
BMI, kg/m^2^	246,506,863	28.0 ± 0.1	62,193,483	33.7 ± 0.1	184,313,379	26.1 ± 0.1	< 0.01
WC, cm	247,113,167	96.1 ± 0.2	62,475,040	111.8 ± 0.3	184,638,127	90.7 ± 0.2	< 0.01
SBP, mmHg	228,354,729	120.3 ± 0.2	58,468,056	130.5 ± 0.3	169,886,673	116.8 ± 0.2	< 0.01
DBP, mmHg	228,354,729	69.6 ± 0.2	58,468,056	74.2 ± 0.3	169,886,673	68.0 ± 0.2	< 0.01
HBA1C, %	244,402,439	5.5 ± 0.0	62,289,095	6.0 ± 0.0	182,113,344	5.4 ± 0.0	< 0.01
FBG, mg/dl	244,721,487	103.5 ± 0.3	62,428,344	119.1 ± 0.6	182,293,144	98.2 ± 0.2	< 0.01
INS, μU/mL	240,197,883	12.5 ± 0.1	61,696,919	20.0 ± 0.3	178,500,964	9.9 ± 0.1	< 0.01
TG, mg/dl	244,131,111	123.4 ± 1.0	62,225,284	186.8 ± 2.6	181,905,828	101.7 ± 0.7	< 0.01
TC, mg/ dL	244,323,493	189.5 ± 0.3	62,260,418	199.2 ± 0.8	182,063,074	186.2 ± 0.4	< 0.01
HDL-C, mg/ dL	244,328,989	53.5 ± 0.4	62,267,715	45.2 ± 0.2	182,061,274	56.3 ± 0.2	< 0.01
LDL-C, mg/ dL	240,007,627	111.8 ± 0.2	59,488,068	118.1 ± 0.6	180,519,559	109.7 ± 0.4	< 0.01
TyG	242,151,586	8.5 ± 0.0	62,178,587	9.1 ± 0.0	179,972,999	8.4 ± 0.0	< 0.01
HOMA-IR	240,175,542	3.4 ± 0.0	61,693,422	6.2 ± 0.1	178,482,120	2.5 ± 0.0	< 0.01
HOMA-IS	240,175,542	0.6 ± 0.0	61,693,422	0.3 ± 0.0	178,482,120	0.7 ± 0.0	< 0.01
HOMA-β	239,926,763	122.9 ± 1.2	61,680,387	151.2 ± 2.3	178,246,376	113.2 ± 1.3	< 0.01

**Table 5:** Baseline characteristics of the adult population after exclusion of drug interference.

Variable	All (N = 22,846)		MetS (N = 5803)		no MetS (N = 17,043)		*P*-value
	WN	Mean ± SE or % (SE)	WN	Mean ± SE or % (SE)	WN	Mean ± SE or % (SE)	
Age,year	195,563,226	45.7 ± 0.2	50,131,040	51.3 ± 0.3	145,432,187	43.8 ± 0.3	< 0.01
Gender	195,563,226		50,131,040		145,432,187		
Male, %		48.1(0.4)		44.7(0.9)		49.2(0.5)	< 0.01
Female, %		51.9(0.4)		55.3(0.9)		50.8(0.5)	< 0.01
Race	195,563,226		50,131,040		145,432,187		
Mexican American, %		8.2(0.5)		8.3(0.7)		8.1(0.5)	0.66
Other Hispanic, %		5.7(0.5)		5.3(0.6)		5.9(0.5)	0.25
Non-Hispanic White, %		68.5(1.0)		72.6(1.2)		67.1(1.0)	< 0.01
Non-Hispanic Black, %		10.7(0.6)		8.8(0.6)		11.4(0.6)	< 0.01
Other Race, %		6.9(0.3)		5.0(0.4)		7.5(0.4)	< 0.01
Education	195,421,651		50,100,525		145,321,126		
< High School, %		16.2(0.5)		19.0(0.7)		15.2(0.5)	< 0.01
High School, %		23.9(0.5)		26.9(0.9)		22.9(0.6)	< 0.01
> High School, %		59.9(0.8)		54.1(1.0)		61.9(0.9)	< 0.01
Smoking	195,423,434		50,075,167		145,348,267		
Yes, %		46.0(0.6)		49.9(1.0)		44.6(0.7)	< 0.01
No, %		54.0(0.6)		50.1(1.0)		55.4(0.7)	< 0.01
Drinking	184,296,904		47,511,065		136,785,839		
Yes, %		73.8(0.7)		67.9(1.0)		75.9(0.7)	< 0.01
No, %		26.2(0.7)		32.1(1.0)		24.1(0.7)	< 0.01
BMI, kg/m^2^	195,094,622	28.4 ± 0.1	49,910,018	33.5 ± 0.1	145,184,604	26.7 ± 0.1	< 0.01
WC, cm	195,563,226	97.5 ± 0.2	50,131,040	111.0 ± 0.3	145,432,187	92.8 ± 0.2	< 0.01
SBP, mmHg	181,122,532	121.3 ± 0.2	46,967,234	130.4 ± 0.4	134,155,298	111.2 ± 0.2	< 0.01
DBP, mmHg	181,122,532	71.1 ± 0.2	46,967,234	75.1 ± 0.3	134,155,298	69.7 ± 0.2	< 0.01
HBA1C, %	195,213,632	5.4 ± 0.0	49,994,240	5.7 ± 0.0	145,219,391	5.3 ± 0.0	< 0.01
FBG, mg/dl	195,519,268	100.3 ± 0.2	50,127,542	110.4 ± 0.4	145,391,725	96.9 ± 0.2	< 0.01
INS, μU/mL	192,331,258	11.7 ± 0.1	49,666,526	18.4 ± 0.3	142,664,733	9.3 ± 0.1	< 0.01
TG, mg/dl	193,768,950	126.6 ± 1.2	50,020,885	184.9 ± 2.9	143,748,065	106.3 ± 0.9	< 0.01
TC, mg/ dL	193,898,027	195.9 ± 0.5	50,046,252	203.6 ± 0.8	143,851,775	193.2 ± 0.5	< 0.01
HDL-C, mg/ dL	193,905,324	54.2 ± 0.2	50,053,549	45.5 ± 0.3	143,851,775	57.3 ± 0.2	< 0.01
LDL-C, mg/ dL	190,408,852	116.8 ± 0.4	47,971,979	122.4 ± 0.6	142,436,873	114.9 ± 0.4	< 0.01
TyG	193,746,609	8.6 ± 0.0	50,017,388	9.1 ± 0.0	143,729,221	8.4 ± 0.0	< 0.01
HOMA-IR	192,308,917	3.0 ± 0.0	49,663,029	5.1 ± 0.1	142,645,889	2.3 ± 0.0	< 0.01
HOMA-IS	192,308,917	0.6 ± 0.0	49,663,029	0.3 ± 0.0	142,645,889	0.7 ± 0.0	< 0.01
HOMA-β	192,186,624	118.5 ± 1.4	49,663,029	152.3 ± 2.1	142,523,595	106.7 ± 1.5	< 0.01

**Table 6:** Baseline characteristics of children and adolescents after exclusion of drug interference.

Variable	All (N = 8458)		MetS (N = 406)		No MetS (N = 8,052)		*P*-value
	WN	Mean ± SE or % (SE)	WN	Mean ± SE or % (SE)	WN	Mean ± SE or % (SE)	
Age group, year	34,511,096		1,688,050		32,823,045		
10–11, %		6.3(0.4)		2.6(1.1)		6.5(0.5)	0.04
12–13, %		22.8(0.7)		16.6(2.9)		23.1(0.8)	0.06
14–15, %		24.1(0.7)		21.0(2.5)		24.3(0.7)	0.23
16–17, %		24.8(0.8)		23.2(3.0)		24.9(0.8)	0.61
18–19, %		22.0(0.8)		36.6(3.4)		21.2(0.8)	< 0.01
Gender	34,511,096		1,688,050		32,823,045		
Male,%		51.3(0.9)		59.2(3.8)		50.9(0.9)	0.04
Female,%		48.7(0.9)		40.8(3.8)		49.1(0.9)	0.04
Race	34,511,096		1,688,050		32,823,045		
Mexican American,%		13.2(0.8)		23.4(2.7)		12.7(0.8)	< 0.01
Other Hispanic,%		6.2(0.5)		7.8(1.8)		6.1(0.5)	0.33
Non-Hispanic White,%		58.1(1.4)		52.6(4.0)		58.4(1.4)	0.13
Non-Hispanic Black,%		14.6(0.9)		10.6(1.6)		14.8(0.9)	0.02
Other Race,%		7.9(0.6)		5.6(1.8)		8.0(0.6)	0.25
BMI, kg/m^2^	34,466,190	23.5 ± 0.1	1,688,050	34.1 ± 0.4	32,778,140	23.0 ± 0.1	< 0.01
WC, cm	34,511,096	80.9 ± 0.3	1,688,050	109.8 ± 1.0	32,823,045	79.4 ± 0.3	< 0.01
SBP, mmHg	31,532,215	109.4 ± 0.2	1,564,610	119.2 ± 0.8	29,967,605	108.9 ± 0.2	< 0.01
DBP, mmHg	31,532,215	60.6 ± 0.3	1,564,610	64.1 ± 0.9	29,967,605	60.4 ± 0.3	< 0.01
HBA1C, %	32,312,866	5.2 ± 0.0	1,644,851	5.3 ± 0.0	30,668,015	5.2 ± 0.0	< 0.01
FBG, mg/dl	32,345,661	93.9 ± 0.2	1,644,851	101.4 ± 0.9	30,700,809	93.4 ± 0.2	< 0.01
INS, μU/mL	31,492,294	12.8 ± 0.2	1,637,020	29.7 ± 1.2	29,855,274	11.9 ± 0.2	< 0.01
TG, mg/dl	33,816,279	83.2 ± 1.0	1,688,050	172.3 ± 9.8	32,128,229	78.5 ± 0.8	< 0.01
TC, mg/ dL	33,869,816	158.0 ± 0.5	1,688,050	173.0 ± 2.5	32,181,766	157.3 ± 0.5	< 0.01
HDL-C, mg/ dL	33,868,016	51.9 ± 0.2	1,688,050	37.1 ± 0.6	32,179,965	52.7 ± 0.2	< 0.01
LDL-C, mg/ dL	33,726,925	89.7 ± 0.5	1,631,889	102.9 ± 2.3	32,095,036	89.0 ± 0.5	< 0.01
TyG	31,864,429	8.1 ± 0.0	1,644,851	8.9 ± 0.0	30,219,577	8.1 ± 0.0	< 0.01
HOMA-IR	31,492,294	3.0 ± 0.1	1,637,020	7.6 ± 0.4	29,855,274	2.8 ± 0.0	< 0.01
HOMA-IS	31,492,294	0.5 ± 0.0	1,637,020	0.2 ± 0.0	29,855,274	0.5 ± 0.0	< 0.01
HOMA-β	31,485,679	154.7 ± 2.3	1,637,020	287.8 ± 11.9	29,848,659	147.4 ± 2.2	< 0.01

Continuous variable data are represented as mean ± SE, and categorical variables are represented as % (SE). T-test was used for continuous variables and Chi-square test was used for classification variables.

*MetS* metabolic syndrome, *WN* weighted N, *SE* standard error, *BMI* body mass index, *WC* waist circumference, *SBP* systolic blood pressure, *DBP* diastolic blood pressure, *HbA1c* glycosylated hemoglobin, *FBG* fasting blood glucose, *FINS* fasting insulin, *TG* triglyceride, *TC*, cholesterol, *HDL-C* high density lipoprotein cholesterol, *LDL-C* low density lipoprotein cholesterol, *TyG* triglyceride-glucose, *HOMA-IR* homeostasis model assessment of insulin resistance, *HOMA-IS* homeostasis model assessment of insulin sensitivity, *HOMA-β* homeostasis model assessment of beta-cell function.

### Predictive ability

**Figure 2:**
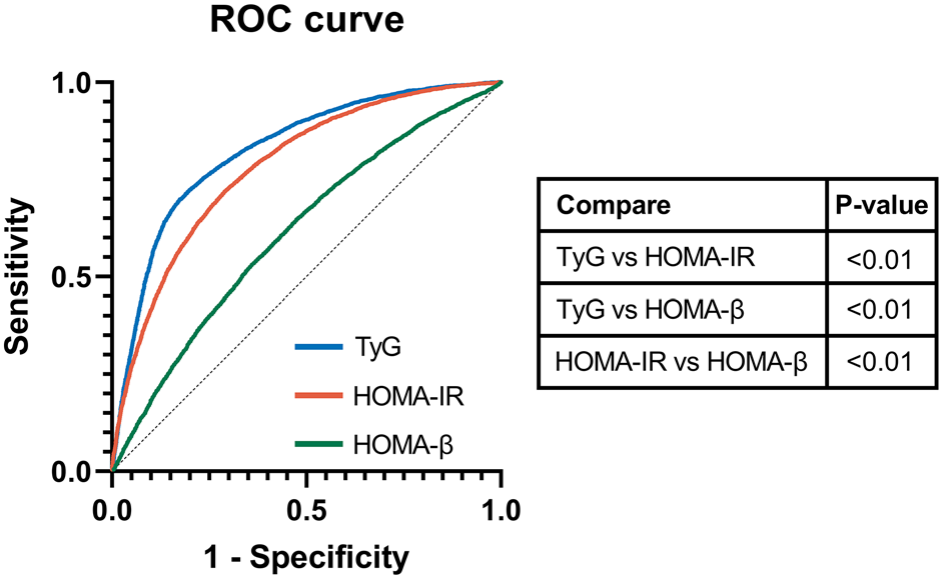
ROC curve for TyG index, HOMA-IR, and HOMA-β for predicting MetS. Abbreviations: ROC, receiver operating characteristic; TyG, triglyceride-glucose; HOMA-IR, homeostasis model assessment of insulin resistance; HOMA-β, homeostasis model assessment of beta-cell function; MetS, metabolic syndrome.

The predictive abilities of the TyG index and HOMA for the diagnosis of MetS in the included population were further evaluated with MetS as a positive diagnosis result. ROC curves were drawn and AUCs were calculated using the TyG index, HOMA-IR, and HOMA-β. The AUC of the TyG index was 0.827 (sensitivity = 71.9%, specificity = 80.5%), and the cutoff was 8.75, which was slightly higher than that of HOMA-IR (0.784) and HOMA-β (0.614), all *P* < 0.01 (Fig. 2). The prevalence of MetS in the population calculated using the TyG index cutoff value was 30.9%, which was higher than that reported in the IDF diagnostic criteria (23.1%). In the adult population, the AUC of the TyG index was 0.796 (sensitivity = 69.8%, specificity = 78.5%) and the cutoff was 8.80, and the prevalence of MetS in the adult population calculated using the TyG index cutoff value was 34.1%. In the child and adolescent population, the AUC of the TyG index was 0.874 (sensitivity = 76.3%, specificity = 87.3%) and the cutoff was 8.62. The prevalence of MetS in the child and adolescent population calculated using the TyG index cutoff value was 16.4%.

### Logistic regression analysis

**Figure 3:**
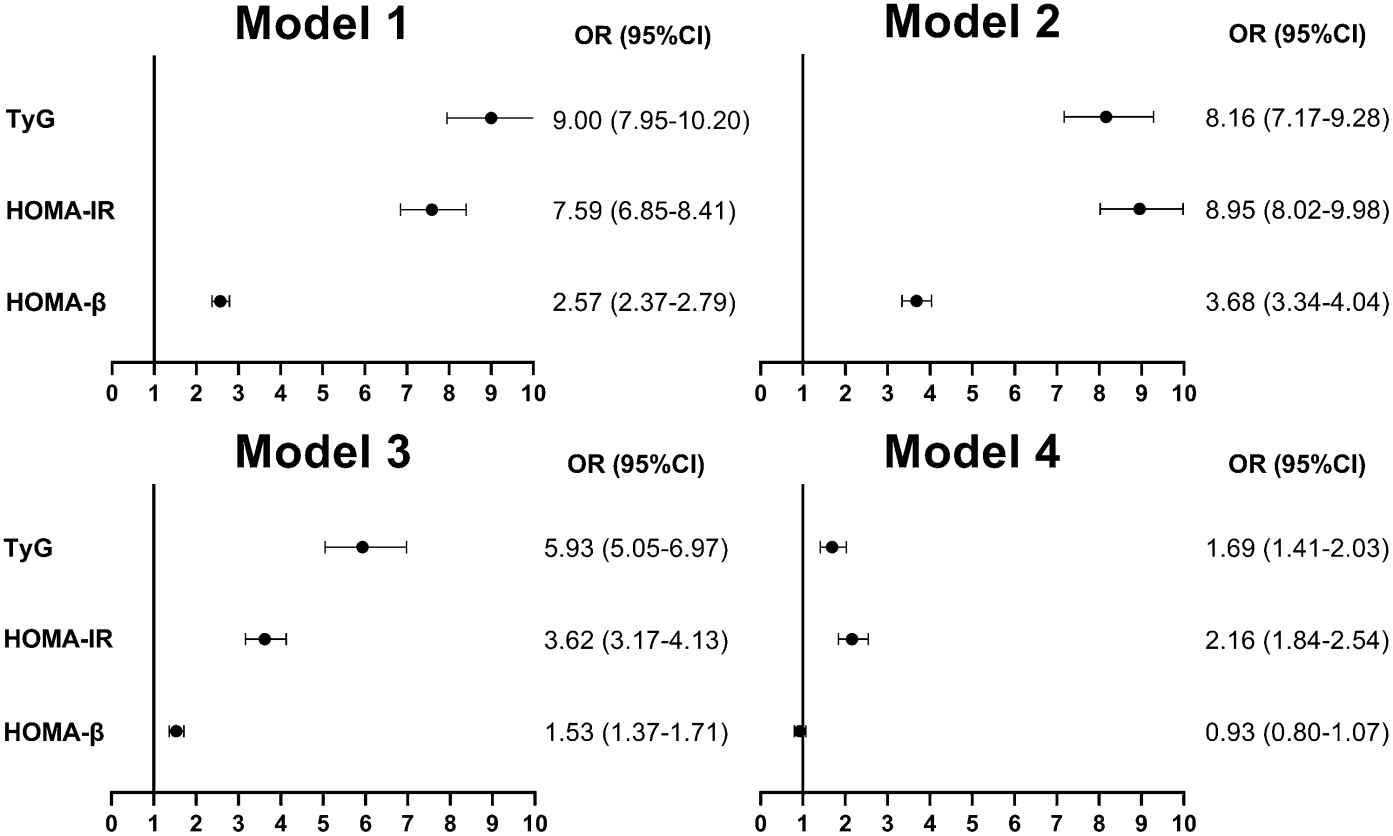
Univariate and multivariate logistic regression forest plots of adjusted factors and the risk for MetS. Note: Model 1: No covariates were adjusted. Model 2: Gender, age, and race were adjusted. Model 3: Age, sex, race, BMI, WC, SBP, and DBP were adjusted. Model 4: Age, sex, race, BMI, WC, SBP, DBP, HbA1c, TC, HDL-C, and LDL-C were adjusted. Abbreviations: MetS, metabolic syndrome; TyG, triglyceride-glucose; HOMA-IR, homeostasis model assessment of insulin resistance; HOMA-β, homeostasis model assessment of beta-cell function; OR, odds ratio; CI, confidence interval, BMI, body mass index, WC, waist circumference, SBP, systolic blood pressure; DBP, diastolic blood pressure; HbA1c, glycosylated hemoglobin; TC, total cholesterol; HDL-C, high-density lipoprotein cholesterol; LDL-C, low-density lipoprotein cholesterol.

After the data were weighted, the TyG index, HOMA-IR, and HOMA-β were transformed into dichotomic variables as independent variables to construct Model 1. Univariate logistic regression analysis showed that high TyG index, HOMA-IR, and HOMA-β were correlated with the risk of MetS. Based on Model 1, the demographic variables (gender, age, and race) were adjusted to construct Model 2. Based on Model 2, the examined variables (body mass index, waist circumference, SBP, and DBP) were adjusted to construct Model 3. Based on Model 3, the laboratory variables (HbA1c, cholesterol, high-density lipoprotein cholesterol, and low-density lipoprotein cholesterol levels) were adjusted to construct Model 4. Multivariate logistic regression analysis showed that, except for Model 4, elevated HOMA-β showed no association with the risk of MetS (odds ratio [OR] {95% confidence interval [CI]}) = 0.93 (0.80–1.07)]. However, in other models, elevated TyG index, HOMA-IR, and HOMA-β were all associated with the risk of MetS, as shown in Fig. 3.

### Subgroup analysis

**Figure 4:**
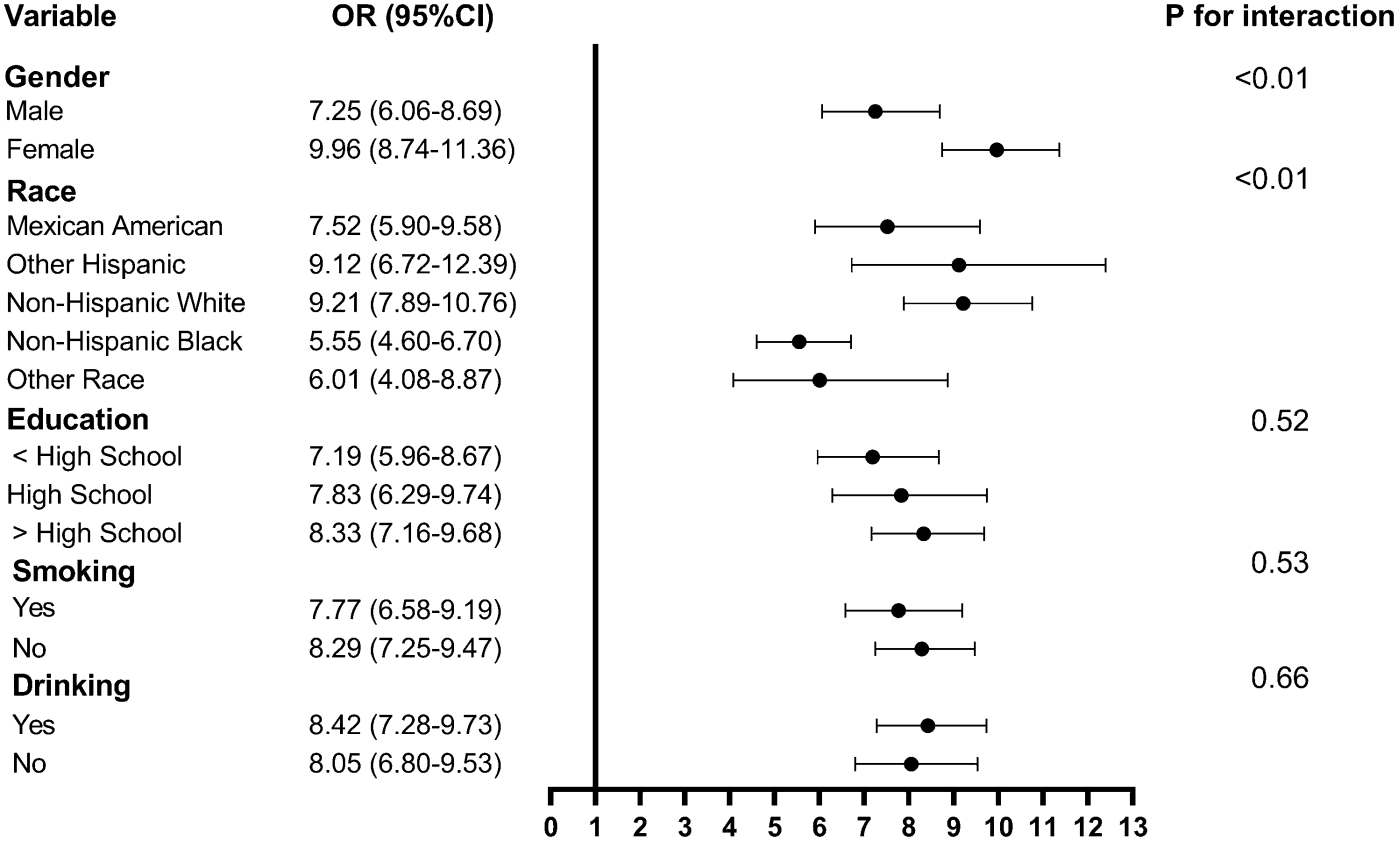
Subgroup analysis of the TyG index in predicting MetS in the adult population. Abbreviations: TyG, triglyceride-glucose; MetS, metabolic syndrome; OR, odds ratio; CI, confidence interval.

Data were weighted for subgroup analysis and the TyG index was transformed into a binary classification variable and taken as exposure variables. Univariate analysis of the adult population revealed that the TyG index could differently predict MetS based on different genders and races (all P-interactions < 0.01). However, there was no difference in the ability of the TyG index to predict MetS among different levels of education, smoking, and drinking (all P-interactions > 0.05), as shown in Fig. 4. For the subgroup analysis of the child and adolescent population based only on gender and race, univariate analysis revealed no differences between gender (P-interaction = 0.45) and ethnicity (P-interaction = 0.29) in the ability of the TyG index to predict MetS.

## Discussion

The strength of this study is that it is the largest investigation to date that has explored the predictive ability of the TyG index and HOMA for MetS within a population sample. NHANES employs a complex, stratified, and random sampling methodology to ensure accurate and reliable test results, thereby securing data authenticity and allowing for generalization to the entire United States population. Our research findings suggest a slightly superior predictive capability of the TyG index over HOMA for MetS.

Although the hyperinsulinemic euglycemic clamp technology is currently the internationally recognized gold standard to evaluate insulin resistance, its implementation requires special equipment and skilled technicians, which is expensive and time-consuming. Moreover, multiple blood samples are required for the test, which can be difficult for patients to accept. Therefore, it is only used for scientific research and cannot be applied in clinical practice on a large scale^14^. HOMA is now commonly used to evaluate insulin function in a clinical setting. HOMA was first proposed by Turner’s research team at Oxford University in 1985^9^. It is a mathematical model established to reflect the interaction of glucose and insulin in different organs (including the pancreas, liver, and surrounding tissues). The model assessed insulin resistance (HOMA-IR) and islet β-cell function (HOMA-β) only by using FBG and fasting insulin values. However, in recent years, a simpler and low-cost test method, namely the TyG index, has been widely used in clinical practice to evaluate insulin resistance. The TyG index was first proposed by scholars in South America. This index can be obtained by determining routine fasting triglyceride and FBG levels in the serum. In 2008, Simental-Mendia et al. used the TyG index for the community screening of healthy participants to check for insulin resistance^8^. Apart from reflecting insulin resistance, studies have shown that the TyG index can also be used as an indicator to predict risks of the chronic complications of diabetes^15,16^, cardiovascular disease^17^, and cerebrovascular disease^18^, among others. Meanwhile, the ability of the TyG index to evaluate MetS has attracted much attention in recent years^2–4^.

Triglyceride levels are typically determined using enzymatic, chromatographic, or chemical methods. Among these, the enzymatic method is the most commonly used and relatively simple procedure. In our study, the triglyceride measurements extracted from the NHANES database were also analyzed using the enzymatic method. This method can be conducted relatively quickly and is suitable for handling large numbers of samples; thus, it is widely used in clinical practice. In contrast, the determination of insulin requires immunological methods such as enzyme-linked immunosorbent assay or radioimmunoassay, which involve more sample-processing steps and experimental procedures. These methods require antibodies to bind to the substance to be measured (such as insulin), followed by the subsequent detection of the conjugated product using a marker such as an enzyme or a radioisotope^19^. Sample processing is relatively complex, especially in remote primary care facilities with limited medical resources, and insulin determination may therefore be difficult. In addition, from the point of cost-effectiveness, the TyG index only requires the detection of glucose and triglyceride concentrations, which is an inexpensive process. On the other hand, HOMA requires the detection of glucose and insulin concentrations, which are associated with higher costs^20^. Therefore, the TyG index is comparatively easier to obtain than HOMA. Moreover, HOMA-β cannot be calculated when fasting glucose levels are less than or equal to 3.5 mmol/ L. We found that the TyG index had a slightly higher AUC value compared with HOMA based on the comparison of the AUC under the ROC curve, suggesting that the TyG index has more advantages in predicting MetS. A higher AUC means that the TyG index has a better predictive ability compared with HOMA, and the sensitivity and specificity of the TyG index are also high. Therefore, these findings suggest that the TyG index may be more suitable for the early screening and prediction of MetS than HOMA, which is, therefore, worthy of further evaluation and application in a clinical setting.

At the same time, our study also found that the cutoff value of the TyG index as a predictor of MetS was 8.75 and that there was little difference in the cutoff value between the adult population and the child and adolescent population. It is noteworthy that the prevalence of MetS calculated using the TyG index cutoff values were all higher than that diagnosed using the IDF criteria across different groups, namely, the total population (30.9% vs. 23.1%), adults (34.1% vs. 29.1%), and children and adolescents (16.4% vs. 4.8%). The difference between the total population and the adult population was within 10%, suggesting that using the TyG index may identify individuals at risk for insulin resistance or early metabolic abnormalities even if they do not meet the IDF criteria. Therefore, using the TyG index may potentially identify patients with MetS at an earlier stage, providing early intervention opportunities to prevent the development of related complications. However, in the child and adolescent population, the difference in the prevalence of MetS calculated using the IDF diagnostic criteria versus the TyG index cutoff values was about 12%, a difference that could significantly increase the workload of pediatricians. This finding suggests that the TyG index may not be appropriate for identifying MetS in children and adolescents. Therefore, additional prospective longitudinal studies are needed to explore the applicability of the TyG index in this specific population.

Using univariate and multivariate logistic regression analyses, we found that high TyG index, HOMA-IR, and HOMA-β values were significantly correlated with the risk of MetS. Even after gradually adjusting for the demographic variable and examination variable and establishing multivariable Model 2 and Model 3, respectively, high TyG index, HOMA-IR, and HOMA-β values still maintained the correlation with the risk of MetS. However, when the laboratory variables were adjusted based on Model 3 and the multivariable Model 4 was established, high HOMA-β was not associated with the risk of MetS because the 95% CI was 1, likely suggesting that HOMA-β had insufficient robustness in predicting MetS. However, in Model 4, high TyG index and HOMA-IR still maintained the correlation with the risk of MetS, providing a solid foundation for the effectiveness of the TyG index and HOMA-IR in predicting MetS.

Subgroup analysis of the TyG index to predict MetS ability showed interactions between genders and races in the adult population. These findings suggested that the applicability of the TyG index in predicting MetS is influenced by different population characteristics. Our study found that the TyG index was slightly better in predicting MetS in female adults than in male adults. There are significant differences in metabolic and physiological characteristics between males and females, including lipid-metabolism patterns, fat distribution, and hormone levels. In general, sex hormone levels vary greatly between men and women, which not only affect fat distribution but also affect metabolism. Androgens are generally associated with more fat accumulation in the abdomen, whereas estrogens may influence fat accumulation in the buttocks and thighs^21^. Additionally, there are differences in basal metabolic rates between men and women, and men usually have a higher basal metabolic rate^22^. Such metabolic and physiological differences may lead to gender differences while predicting MetS. Our study found that the ability of the TyG index to predict MetS also differed among adults from different races; however, this difference was not significant. Differences in metabolic characteristics, genetics, lifestyle, and culture among ethnic groups may contribute to differences in body fat and metabolism^23^. Therefore, gender and ethnic differences in the adult population may need to be considered during the practical application of the TyG index in predicting MetS to develop more personalized prevention and intervention measures.

We acknowledge certain limitations of our study. First, due to the wide time span, variations in laboratory methods or locations for measuring glucose, insulin, and triglycerides across different cycles might have potentially influenced our results. Second, being a cross-sectional study derived from observational surveys, only associations and not causes could be established.

## Conclusions

The TyG index is a convenient and easy-to-calculate parameter in clinical practice. Using NHANES data, we found that TyG still had a high diagnostic value for MetS. It even slightly outperformed the traditional HOMA and the cutoff was 8.75. The prevalence of MetS in the population calculated using the TyG index cutoff was higher than that reported in the IDF diagnostic criteria. This may help identify more cases of MetS, but as the prevalence differs widely when calculating the population of children and adolescents, the TyG index that identifies MetS may not apply to this particular population. In logistic regression analysis, after adjusting for multiple variables, the TyG index still maintained a correlation with the risk of MetS. Subgroup analysis of the adult population indicated differences in the ability of the TyG index to predict MetS in different genders and races, which may need to be considered prior to practical applications. In conclusion, the TyG index may be used to predict MetS in a clinical setting, thereby serving as an important reference for the early prevention of and intervention for MetS. The findings of our study highlight the use of this important index in future clinical practice.

## Data availability

All databases are accessible through the NHANES website (https://wwwn.cdc.gov/nchs/nhanes/Default.aspx).

### Supplementary Information

Supplementary Information.
