# Supplementary material for: Superiority of the triglyceride glucose index over the homeostasis model in predicting metabolic syndrome based on NHANES data analysis
Source: Sci Rep. 2024 Jul 5;14:15499. doi: 10.1038/s41598-024-66692-9 (PMC11226440; doi:10.1038/s41598-024-66692-9)
Supplement: Supplementary file 1 — Supplementary Information. [file 41598_2024_66692_MOESM1_ESM.docx]

**Supplementary Table S1** Waist circumference percentile regression values in the United States for children aged 10-18 years and adolescents combined, according to sex

| **Age (year)** | **Percentile for boys** | | | | | **Percentile for girls** | | | | |
| --- | --- | --- | --- | --- | --- | --- | --- | --- | --- | --- |
|  | **10^th^** | **25^th^** | **50^th^** | **75^th^** | **90^th^** | **10^th^** | **25^th^** | **50^th^** | **75^th^** | **90^th^** |
| 10 | 57.0 | 59.8 | 63.3 | 69.2 | 78.0 | 56.3 | 58.6 | 62.8 | 68.7 | 76.6 |
| 11 | 58.7 | 61.7 | 65.4 | 71.7 | 81.4 | 57.9 | 60.3 | 64.8 | 71.1 | 79.7 |
| 12 | 60.5 | 63.5 | 67.4 | 74.3 | 84.8 | 59.5 | 62.0 | 66.7 | 73.5 | 82.7 |
| 13 | 62.2 | 65.4 | 69.5 | 76.8 | 88.2 | 61.0 | 63.7 | 68.7 | 75.9 | 85.8 |
| 14 | 63.9 | 67.2 | 71.5 | 79.4 | 91.6 | 62.6 | 65.4 | 70.6 | 78.3 | 88.8 |
| 15 | 65.6 | 69.1 | 73.5 | 81.9 | 95.0 | 64.2 | 67.1 | 72.6 | 80.7 | 91.9 |
| 16 | 67.4 | 70.9 | 75.6 | 84.5 | 98.4 | 65.7 | 68.8 | 74.6 | 83.1 | 94.9 |
| 17 | 69.1 | 72.8 | 77.6 | 87.0 | 101.8 | 67.3 | 70.5 | 76.5 | 85.5 | 98.0 |
| 18 | 70.8 | 74.6 | 79.6 | 89.6 | 105.2 | 68.9 | 72.2 | 78.5 | 87.9 | 101.0 |

Data from the International Diabetes Federation. IDF consensus definition of metabolic syndrome in children and adolescents. Available from: <https://idf.org/media/uploads/2023/05/attachments-31.pdf>. Accessed January 11, 2024.
